# Congenital Melanocytic Nevus with Neurocristic Cutaneous Hamartoma: A Case Report

**DOI:** 10.3390/dermatopathology12020012

**Published:** 2025-04-10

**Authors:** Dina El-Rayes, Katlin Wilson, Sheilagh Maguiness, Daniel Miller, Gerardo Cazzato, Alessio Giubellino

**Affiliations:** 1 Department of Laboratory Medicine & Pathology, University of Minnesota, Minneapolis, MN 55455, USA; delrayes@umn.edu (D.E.-R.); wils2669@umn.edu (K.W.); 2 Department of Dermatology, University of Minnesota, Minneapolis, MN 55455, USA; smaguine@umn.edu (S.M.); mill0581@umn.edu (D.M.); 3 Section of Molecular Pathology, Department of Emergency and Organ Transplantation (DETO), University of Bari “Aldo Moro”, 70124 Bari, Italy; gerardo.cazzato@uniba.it

**Keywords:** congenital melanocytic nevus, nodular proliferative neurocristic cutaneous hamartomas, Schwannian differentiation

## Abstract

Congenital melanocytic nevi (CMN) are benign tumors present at birth or arising in the first few months of life. A small subset of these nevi present with mild atypical features and heterogeneous differentiation, including Schwannian differentiation. We present a case of a 3-week-old with a 7 cm red/purple scalp nodule consistent with CMN with mild atypical heterogeneous areas. On histology, there were dermal nests of spindle cells in a fibrillar matrix, with increased vessels and clusters of small round melanocytes interspersed between collagen bundles and around adnexal structures. The lesion also exhibited rare pagetoid ascent of melanocytes as single cells and nests. Overall, these features were consistent with a CMN with nodular proliferative neurocristic cutaneous hamartoma (NCH) with a component of a compound mild atypical melanocytic proliferation. Next generation sequencing (NGS) identified a novel SH2B1::BRAF fusion. This case highlights the diagnostic challenges of heterogeneous differentiation within CMN in young children.

## 1. Introduction

Congenital melanocytic nevi (CMN) are pigmented cutaneous lesions that can arise during the neonatal period and appear at birth or arise shortly after birth. When CMN begin to develop in utero, they can potentially arise as early as the third and fourth weeks of gestation [1,2], with precursor melanocytes migrating from the neural crest during early embryogenesis [3]. During this process, CMN may acquire mutations, most frequently in the NRAS gene, resulting in overproliferation of melanocytes, yielding a range of clinical presentations [4]. The size-based classification of CMN includes small (<1.5 cm in greatest dimension), medium (1.5–19.9 cm in greatest dimension), and large/giant (≥20 cm in greatest dimension in projected adult size) [5]. The large/giant CMN are more associated with an increased risk of malignant transformation [6,7].

Although CMN are generally considered common, GCN are rarer, with an estimated occurrence ranging from 1 per 20,000–500,000 live births [8]. While most GCN remain benign, approximately 6% of GCN are thought to develop melanoma at the site of the nevus [6]. Consequently, accurately distinguishing atypical CMN from melanoma in neonates is of paramount importance, given that CMN is a known risk factor for childhood melanoma; however, melanoma remains rare in newborns [9].

Recent molecular investigations have substantially advanced our understanding of CMN pathogenesis. While BRAF V600E mutations are classically associated with acquired nevi and melanoma, mutations in NRAS, particularly affecting the NRAS/MAPK pathway, are frequently detected in CMN [10,11]. These mutations lead to persistent activation of the MAPK pathway, which drives melanocyte proliferation [11].

Building on this, neurocristic cutaneous hamartoma (NCH) is recognized as a rare, benign tumor of the skin and superficial soft tissue, postulated to originate from persistently active pluripotent cells derived from the neural crest [12,13]. Due to overlapping histopathological features, properly identifying NCH and differentiating it from melanoma or other melanocytic lesions can be challenging, and the limited literature available further complicates the comprehensive study of this entity.

We herein describe a neonate presenting with a CMN with NCH that demonstrates mild atypical histopathologic features, Schwannian differentiation, and a novel SH2B1::BRAF fusion. In addition, we provide a comprehensive review of the literature, highlighting histopathology, immunohistochemical markers, molecular alterations, and management strategies for NCH.

## 2. Case Presentation

**Figure 1:**
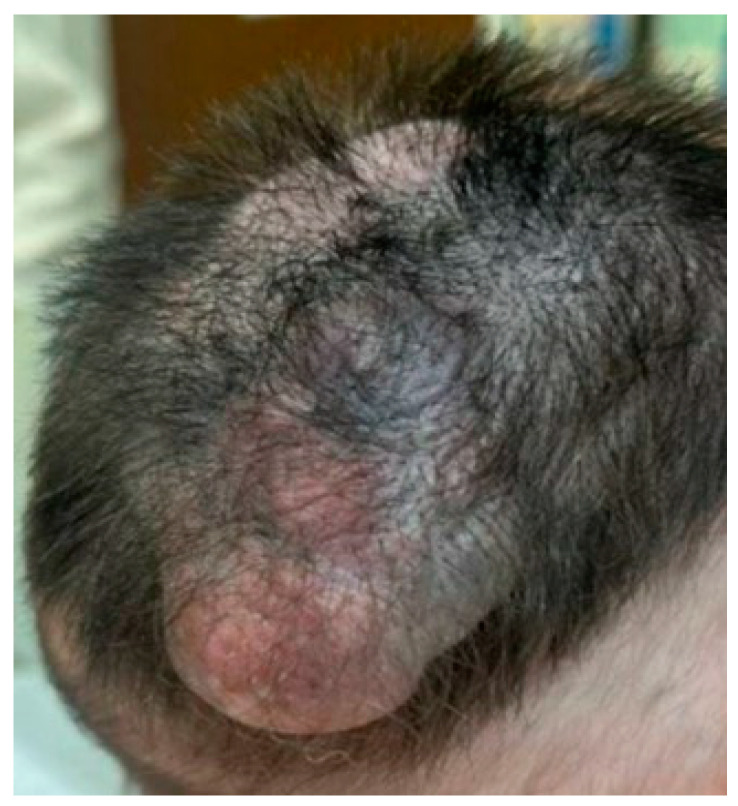
Patient with a proliferative nodule with hairy surface arising in CMN on the scalp. This photo was taken at age of 9 months during follow-up where the lesion size increased to 8.5 cm.

A 3-week-old neonate underwent clinical evaluation for a CMN measuring 7 cm in greatest diameter, located on the right vertex and extending towards the occipital and parietal regions of the scalp. The lesion was non-tender, soft, and demonstrated heterogeneous areas of red/purple discoloration with hair growth (Figure 1).

**Figure 2:**
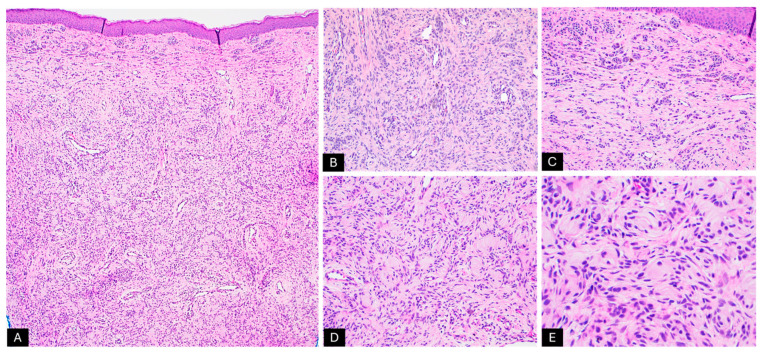
Histopathologic examination by H&E stain; (**A**–**C**) Involvement of the dermis predominantly by spindle-shaped Schwannian cells and small nests of round melanocytes (40X, 200X, and 200X, respectively); (**D**,**E**) Low-power (200X) and high-power (400X) images of melanocytes forming whorls within a fibrillar matrix.

**Figure 3:**
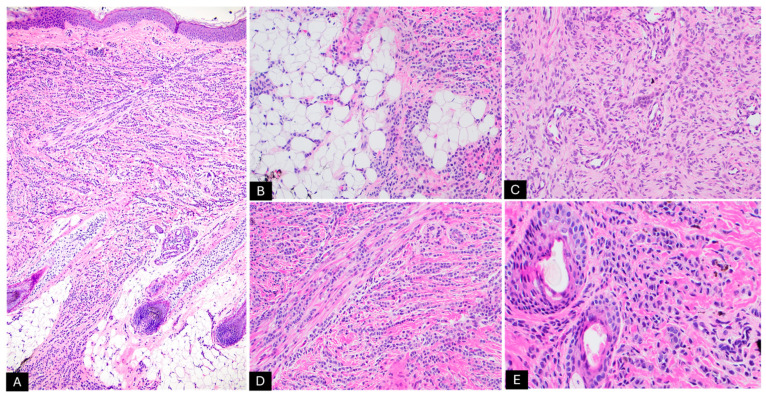
Histopathologic examination by H&E stain; (**A**,**B**) A dense melanocytic proliferation centered in the reticular dermis, extending into the subcutaneous adipose tissue along connective tissue septa (40X and 200X, respectively); (**C**) Increased vascularization within the lesion; (**D**,**E**) The melanocytes dispersed among collagen bundles and surrounding epithelial adnexal structures (200X and 400X, respectively).

**Figure 4:**
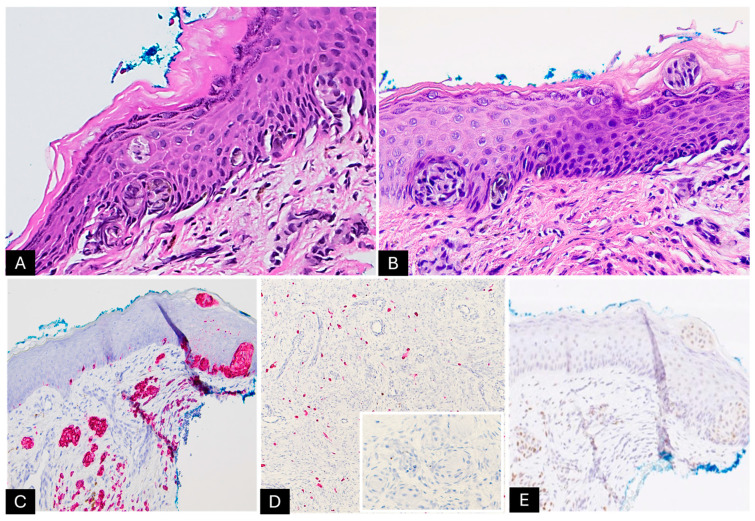
(**A**,**B**) Rare pagetoid arrays of melanocytes, appearing as both individual cells and rounded nests (200X); (**C**,**D**) Melan-A immunostain is strong and diffuse positive in the melanocytic cells and nests while being negative in the Schwannian differentiation areas (100X, 100X, 400X); (**E**) PRAME immunostain is negative (200X).

Histopathological examination of the specimen revealed extensive involvement of the dermis by small nests of spindle-shaped melanocytes (Figure 2A–C). Some of these melanocytes were observed forming whorls within a fibrillar matrix (Figure 2D,E). Increased vascularity was noted, along with clusters of small, round melanocytes interspersed between collagen bundles and around epithelial adnexal structures, extending into the subcutaneous septa and between adipocytes (Figure 3A–E). The lesion also exhibited rare pagetoid arrays of melanocytes as both single cells and round nests, with pale, vacuolated cytoplasm and dusty melanin (Figure 4A,B). We did not identify any alarming features, such as increased mitotic activity or perineural invasion. Melan-A immunohistochemical stain was diffusely positive in the melanocytic component, however, it was negative in the areas with Schwannian differentiation (Figure 4D), and PRAME staining was negative (Figure 4E). These histopathological findings were interpreted as NCH with Schwannian differentiation and focal mild atypical features within a CMN, highlighting the complexity of this lesion.

Next generation sequencing (NGS) assay identified a novel SH2B1::BRAF gene fusion in our case. While BRAF V600E mutations are well-documented in CMN [14], the occurrence of BRAF fusions in NCH within CMN is not documented in the literature, to our knowledge, and their biological significance in congenital lesions is not yet fully understood.

As of 11 months post-diagnosis, our patient remains in good clinical condition, with no evidence of local transformation, metastatic spread, or other concerning prognostic features. These observations continue to suggest a sustained favorable prognosis of NCH.

## 3. Review of Literature

A total of 29 studies detailing cases that fulfill the criteria for NCH were identified. Table 1 describes the clinical characteristics, diagnostic approaches, and molecular findings based on these studies.

The term “pilar neurocristic hamartoma” was historically used to describe lesions now more broadly recognized as “cutaneous neurocristic hamartoma” (NCH) [15]. NCH within a CMN represents a relatively uncommon variant of melanocytic proliferation. These lesions are thought to originate from neural-crest-derived cells, exhibiting differentiation along melanocytic and Schwannian lineages [12,16,17,18,19]. Although NCH has been described in the existing literature, such reports are limited, and the biological behavior, genetic background, and natural history of these lesions are still not fully understood.

Although rare, NCH represents a diagnostically challenging entity due to its complex histopathological features and overlapping characteristics with other melanocytic and neural tumors. Its classification remains controversial, with multiple perspectives in the literature. Some studies describe NCH as a lesion exhibiting fibrogenic, melanocytic, and neurosustentacular differentiation, reflecting its complex histogenesis [12]. Others consider it a melanocytic tumor composed of a mosaic of areas resembling congenital epithelioid melanocytic nevus, neurofibroma, schwannoma, and blue, cellular, and epithelioid blue nevus (BN) [20]. Another viewpoint classifies NCH as a variant of cellular BN with a prominent Schwannian component due to overlapping histopathological features [21]. Additionally, some authors propose that NCH represents a variant of patch- or plaque-type BN or a combined nevus with blue-nevus-like features [22]. Given these differing viewpoints, NCH is often regarded as a distinct entity within the spectrum of neural-crest-derived lesions, though its precise classification remains a subject of ongoing debate.

NCHs typically manifest as asymptomatic, well-circumscribed, elevated cutaneous nodular proliferations that exhibit variations in size and coloration, predominantly located on the scalp and torso [1,12,16,17,19,23,24,25,26,27,28,29,30,31]. NCH can manifest with various hair-related alterations, including focal alopecia or increased hair growth [27,32,33]. Beyond these changes, poliosis has also been observed in related neural-crest-derived conditions and warrants consideration. Poliosis arises from a diminished or absent production of melanin within hair follicle melanocytes and has been described in the context of hereditary disorders, inflammatory processes, medication use, and neoplastic lesions [19,26]. In CMN, poliosis appears to result from the arrest of neural crest cells within hair follicles and, accordingly, it is reasonable to propose a similar developmental mechanism in cases of poliosis reported in NCH [19].

Notably, in only two studies in the literature did NCH an unusual presentation as cutis verticis gyrata, a condition defined by the presence of deep, convoluted furrows and folds on the scalp that closely resemble the surface of the brain [27,33]. Histologically, NCH is characterized by spindle-shaped or occasionally polygonal cells arranged in whorls or nodules set in a fibrillar or myxoid stroma [8,12,16,19,23,29,34]. Increased vascularity may occasionally be observed in NCH, with mitoses generally infrequent to absent in benign lesions; significant mitotic activity or perineural invasion warrants careful evaluation for potential malignant transformation or alternative diagnoses [6,8,17,28,32,34,35]. Although NCH primarily involves the dermis, in rare cases, it may extend deeply into the subcutaneous tissue, even infiltrating bone marrow spaces [31,36].

Immunohistochemical stains are crucial for diagnosing NCH. The melanocytic components of NCH typically express markers such as Melan-A and HMB-45, in addition to S-100, the latter suggesting a neurocristic origin [16,18,19,23,24,25,32,34]. On the other hand, the neural areas with Schwannian differentiation are negative for Melan-A and HMB-45 but show S-100 reactivity and may also show co-expression of other nerve sheath markers [16,18,19,23,24,25,30,34]. Increased CD34 staining has been observed in the surrounding stroma of these lesions, which can help distinguish them from other entities [25]. These combined findings reflect lineage plasticity in neural-crest-derived cells and highlight the complex histogenesis of NCH.

Clinically and microscopically, diagnosing NCH can present a notable challenge due to its shared features with other dermal melanocytic neoplasms, such as proliferative nodules in CMN, BN, or early melanoma arising within a CMN [24,27,31,33,37]. Proliferative nodules often show architectural disorder and increased cellularity, features that may overlap with NCH [30]. Additionally, the presence of a Schwannian component in NCH adds another layer of complexity, potentially mimicking neural tumors or combined neurocristic proliferations. Distinguishing NCH from melanoma requires a careful morphologic evaluation supported by immunohistochemical stains and, in select cases, molecular analyses. The absence of established molecular drivers associated with melanoma such as BRAF V600E mutations, along with the lack of PRAME staining expression by immunohistochemistry, a marker that is often positive in melanoma, can help confirm a benign diagnosis in NCH [18,28,30].

Most reported cases of NCH lack the genetic aberrations frequently observed in melanoma or other atypical proliferations with malignant potential. While CMN commonly harbor NRAS mutations and rarely BRAF V600E mutations, NCH has not been linked to consistent molecular alterations [9,10,11,28]. Although occasional genetic abnormalities such as atypical gene fusions or rare mutations may be identified, these do not necessarily impart aggressive biological behavior. When compared to malignant melanoma, NCH showed relative overexpression of IGF2 and H19, suggesting the abnormal gene imprinting and IGF2 overexpression are likely to play crucial rules in the development of NCH [13]. To date, there have been no large-scale genomic studies systematically evaluating NCH, and most knowledge is derived from single case reports or small series [13,18,28,29,30]. Unlike CMN, which presents no apparent chromosomal abnormalities, NCH can demonstrate multiple chromosomal gains and losses as detected by comparative genomic hybridization and chromosomal microarray analysis [12,18,24,28,29,30].

The existing literature suggests that NCH generally follows a benign course. Conventional CMN carries a variable, though generally quite low, lifetime risk of malignant transformation. The presence of NCH within such lesions does not appear to significantly increase the risk of developing melanoma based on currently available data. Most reported NCH cases show stable clinical behavior, and in some instances, partial regression has been documented over time [8,12,29]. However, to date, there are few studies that have addressed malignant NCH, making such occurrences exceedingly rare [17,23,28,35].

In a review of NCH that progressed to malignancy, it was found that such transformations can occur in both congenital and acquired lesions, among patients ranging from 11 to 67 years old [23]. Malignancies in the congenital lesions developed over a notably longer span compared to acquired ones [23]. Histopathological examinations revealed a deep intradermal or subcutaneous origin of those tumors, which often presented as well-defined, multinodular, melanin-rich proliferations composed of bland, small, rounded to spindled cells [23,35]. Several tumors showed complex patterns such as trabecular or nested arrangements, as well as nuclear palisading and perivascular pseudorosettes. In a few cases, large, pleomorphic epithelioid cells formed the predominant population. Unlike conventional melanomas, these tumors tend to recur as large nodules and may metastasize many years, or even decades, after the initial diagnosis [23,35].

Detailed histopathological assessment remains the cornerstone of diagnosis of this entity. When feasible, immunohistochemical markers such as PRAME can be instrumental in ruling out melanoma [18,30]. Although molecular testing may be considered, its diagnostic value is not fully established due to the sparse genetic data currently available for these lesions.

Management of NCH typically involves close clinical observation, with surgical intervention reserved for lesions that appear ambiguous or pose cosmetic concerns [18,23,35,36]. Familiarity with NCH among dermatologists and dermatopathologists is crucial to prevent both misdiagnosis and unwarranted aggressive treatment. Whenever possible, wide local excision is the treatment of choice, coupled with regular, extended follow-up to ensure patient safety and optimize outcomes [8,16,17,24]. However, in cases where lesions display poorly defined margins, Mohs surgery has been suggested as an alternative approach [38].

## 4. Discussion

The pathogenesis of NCH is fundamentally tied to disrupted developmental processes of neural-crest-derived cells. These cells, which are pluripotent during embryogenesis, migrate throughout the body and differentiate into various cell types, including melanocytes, peripheral neurons, and Schwann cells. In NCH, a disruption in this migration and differentiation process leads to hamartomatous growth. This disruption is presumed to be driven by genetic mutations or epigenetic modifications that affect key cell signaling pathways critical for neural crest cell development, such as the WNT, Notch, and Sonic Hedgehog pathways [39]. Additionally, alterations in the microenvironment may contribute to abnormal cellular behavior. Molecular analyses have revealed both overexpression and mutations of genes in these lesions, promoting unchecked cellular proliferation, survival, and migration, which mimic oncogenic processes [13]. As a result, these tumors frequently show a mixed cellular composition characteristic of their neural crest origin, including elements of melanocytic and nerve sheath tumors. This complex interplay of genetic factors and environmental factors results in the remarkably wide range of morphological and clinical manifestations of NCH, making its pathogenesis a notably rich subject for ongoing research within the dermatological and oncological fields.

Our case demonstrates a particularly rare and diagnostically challenging presentation of NCH arising within a CMN, with a novel molecular finding, the SH2B1::BRAF gene fusion. The lesion exhibited some histopathologic features that might be concerning in terms of malignancy (e.g., biphasic proliferation with a spindle cell component in a fibrillar stroma, increased vascularity, melanocytes demonstrating rare pagetoid spread). Such features may mimic melanoma, particularly in a young patient [8,28]. Pagetoid arrays are commonly associated with melanoma but can also occur in benign lesions, such as CMN in young children [40]. These benign pagetoid melanocytes typically demonstrate minimal to no cytologic atypia [40]. In our case, the pagetoid cells observed lacked significant atypical features, supporting a benign interpretation. Accurately recognizing NCH is essential not only to prevent misclassification as melanoma but also due to the potential for melanomas to develop within these lesions at unpredictable times. The deep location and pre-existing pigmented state of NCH can make such developments difficult to detect.

Further complicating the diagnostic landscape, both NCH and BN share overlapping histopathological characteristics that challenge diagnosis [15]. Clinically, NCH typically presents as relatively large (3–18 cm) blue-gray plaques, frequently located on the scalp, sometimes accompanied by focal alopecia [12,16,23,30,31,32,33,34]. Conversely, blue nevi, including cellular variants, generally manifest as smaller lesions (approximately 1–2 cm), often localized deeper within the dermis [15,21,41]. Histologically, NCH may exhibit overlapping features with BN, particularly plaque-type BN; however, the heterogeneous histologic presentation of NCH and the distribution of melanocytes around hair follicles, eccrine glands, vessels, and nerves and involvement of the subcutaneous tissue favor a diagnosis of NCH [15,23,34,42]. Immunohistochemical staining often reveals diffuse S-100 positivity in both neuroid and melanocytic components of NCH, whereas HMB-45 staining is usually limited to the melanocytic cells [16,18,19,23,25,34]. Similarly, BN with Schwannian differentiation shows spindle-shaped melanocytes forming compact dermal proliferations [21]. The distinction between these entities ultimately relies on a combination of clinical presentation, lesion size, anatomical location, and meticulous histopathological evaluation.

NCHs are believed to result from an aberrant neuromesenchymal process, thereby reflecting the wide-ranging developmental differentiation potential of migrating neural crest cells. Although melanocytic elements often predominate, neurosustentacular and neuromesenchymal components may emerge as principal constituents, and in cephalic lesions, cartilage or muscle derived from the cephalic neural crest may be present [43]. The occurrence of ectopic cartilage within a melanocytic lesion remains highly uncommon [44], and thus far, only a single documented case of cartilage formation has been described in a congenital NCH [43]. When considering the differential diagnosis of NCH, it is also important to carefully differentiate it from blue nevus, as both lesions originate in the dermis and exhibit immunoreactivity for S-100 and HMB-45, markers for neural crest origin and active melanocytes, respectively. However, the primary distinguishing factor is the Schwannian differentiation observed in NCH, which is generally absent in blue nevi. Additionally, NCH typically shows strong CD34 positivity in the stromal cells, while blue nevi do not, providing an additional diagnostic criterion [19]. One of the key mimickers that must be excluded in the differential diagnosis is CMN with neurotization, given the histologic overlap with NCH. However, immunohistochemical staining helps distinguish between these entities. Melan-A shows strong positivity within the neurotized areas, consistent with melanocytic differentiation, whereas the Schwannian component in NCH is entirely negative for Melan-A, supporting its neural (non-melanocytic) origin [16,18,23,24,33,34,45,46,47]. This contrasting staining pattern highlights the biphasic nature of NCH and the importance of using immunohistochemical staining to differentiate it from other mimickers.

NCH can be either congenital or acquired, and either form can undergo malignant transformation or evolve into melanoma, though this remains relatively rare. Acquired NCH demonstrates a somewhat heightened tendency for recurrence and malignant progression when compared to the congenital ones. In a reported series of malignant NCH, the lesion underwent malignant transformation over an extremely varied timeframe, spanning from 1 year to 67 years from the initial diagnosis [23]. In certain instances, the tumor metastasized to vital organs such as lungs, liver, spleen, and lymph nodes [23]. The literature suggests that malignant NCH may be distinct from melanoma based on the lack of GNAQ, NRAS, BRAF, and KIT mutations that are commonly identified in melanoma. This genetic distinction may contribute to a comparatively more favorable clinical course for malignant NCH compared to melanomas originating in GCN or malignant blue nevus.

In this current case, the absence of PRAME expression and the lack of other overt malignant features (including increased mitotic figures and nuclear pleomorphism) supported a benign interpretation. PRAME demonstrates high sensitivity and specificity in the diagnosis of most melanoma variants, and its negativity in this case favored a non-malignant process [12,18,30]. Moreover, the novel SH2B1::BRAF fusion identified by NGS underscores the need for further investigation into the clinical implications of these BRAF fusions in NCH pathogenesis. While NRAS mutations, and rarely BRAF V600E mutations, are well-documented in CMN, fusions involving BRAF are less frequently observed and not fully understood [10]. The significance of this particular fusion in the context of NCH is still uncertain, however, current evidence implies that such lesions do not behave like melanoma, even in the presence of atypical or unusual molecular findings [23].

From a clinical standpoint, recognizing and accurately classifying NCH is imperative. Misdiagnosing such lesions as melanoma could result in unnecessary radical interventions in neonates and children, potentially causing significant scarring or long-term damage.

A thorough histopathological evaluation, augmented by immunohistochemistry (including PRAME) and, when appropriate, molecular testing, can provide substantial reassurance of a benign diagnosis. Such a systematic approach ensures that future management decisions, whether ongoing surveillance or surgical intervention, are guided by accurate pathological interpretation.

## 5. Conclusions

In conclusion, the rare, complex case we are presenting involves NCH within GCN, distinguished by its atypical histological features and a novel SH2B1::BRAF fusion detected by NGS. This case highlights the inherent diagnostic complexities associated with neurocristic proliferations. By integrating the limited, yet gradually expanding, literature on NCH, diagnostic criteria can be refined to support benign interpretations in challenging cases. The importance of comprehensive morphological, immunohistochemical, and molecular assessments is highlighted in accurately diagnosing NCH and distinguishing it from other melanocytic lesions. As more cases of NCH are documented in the literature, our collective understanding of its pathogenesis and clinical behavior is expected to deepen significantly. This enhanced understanding will undoubtedly contribute to greater diagnostic accuracy and better-informed patient care decisions in the future.

## Figures and Tables

**Table 1:** This table summarizes clinical, histologic, immunohistochemical, molecular, prognostic, malignant transformation, and management findings in neurocristic hamartomas (NCHs), including cases arising within congenital melanocytic nevi (CMN), de novo NCH, and malignant NCH, based on an extensive review of the literature.

Category	Summarized Findings
Clinical Findings	- Usually congenital or early-onset - Variable pigmentation (often less pigmented or hypopigmented than conventional CMN) - Variable size (often > 3 cm) - Commonly scalp and head involvement, but can involve trunk and limbs - Associated findings in some cases: poliosis, alopecia, cutis verticis gyrata-like appearance, or skeletal involvement
Histologic Findings	- Epidermal and dermal proliferation of melanocytes - Schwannian differentiation - Lesions can be either predominantly melanocytic or neuromesenchymal - Myxoid or collagenous background - Increased vascular proliferation - Generally low mitotic rate unless malignant transformation occurs - Usually extends deep into the subcutaneous tissue
Immunohistochemistry	- Positive: S100, SOX10, Melan-A, and HMB-45 - Negative: PRAME - Areas with Schwannian differentiation: positive for S100 and EMA, while negative for Melan-A and HMB-45 - Stromal cells: positive for CD34
Molecular Features	- Multiple chromosomal gains and losses by comparative genomic hybridization and chromosomal microarray analysis - No mutations identified by molecular analyses
Prognosis	- Generally benign behavior, particularly in smaller lesions - Rare malignant transformations reported; prognosis guarded in these cases - Larger lesions warrant closer follow-up
Malignant Transformation Findings	- Clinical signs: rapid growth - Histology: increased cytologic atypia, mitotic figures, invasion of deeper tissue structures - Molecular changes: acquisition of additional genetic alterations
Management	- Conservative observation for small lesions - A wide local excision is considered for cosmetic or symptomatic relief, especially with lesion growth or concerning clinical/histologic features - Close long-term follow-up recommended - Mohs surgery for poorly defined margins

## Data Availability

Data are contained within the article.

## Abbreviations

The following abbreviations are used in this manuscript:

BN	Blue nevus
CMN	Congenital melanocytic nevus
GCN	Giant congenital nevus
NCH	Nodular proliferative neurocristic cutaneous hamartoma
NGS	Next generation sequencing
